# Results of Neuropathy Screening Test for Lower Limb Amputees With Diabetes Mellitus and Their Prosthetic Rehabilitation: A Cross-Sectional Study

**DOI:** 10.7759/cureus.40352

**Published:** 2023-06-13

**Authors:** Yohei Tanaka, Takaaki Ueno

**Affiliations:** 1 Rehabilitation Medicine, JR Tokyo General Hospital, Tokyo, JPN

**Keywords:** diabetes mellitus, physical therapy, orthoses, prosthetic rehabilitation, diabetic neuropathy, the ipswich touch test, lower limb amputees with diabetes

## Abstract

Introduction

To prevent foot ulcers and subsequent amputation on the non-amputated side, we conducted tests for diabetic neuropathy. The results were then used in prosthetic rehabilitation for lower limb amputees with diabetes mellitus.

Materials and methods

This cross-sectional retrospective study included patients admitted to our convalescent rehabilitation ward for prosthetic rehabilitation between April 2019 and December 2022 following lower limb amputation due to diabetes. We investigated the positive rate of the modified Ipswich Touch Test (mIpTT) in lower limb amputees with diabetes, and physical and orthotic therapy and prosthetic rehabilitation based on medical records.

Results

Twenty-seven transtibial amputees and nine transfemoral amputees had diabetes. The mIpTT results were positive in 22 (81%) transtibial and eight (89%) transfemoral amputees. There were no apparent differences in positivity rates by amputation level, gender, or age. Based on these results, personalized physical therapy and insoles were prescribed for the non-amputee foot in parallel with prosthetic rehabilitation.

Conclusions

Lower limb amputees with diabetes have diabetic neuropathy more frequently than diabetic patients without amputation. As a result, they may also be at a higher risk of developing foot ulcers and subsequent amputation due to neuropathy. Therefore, rehabilitation to prevent amputation on the non-amputated side of lower limb amputees with diabetes had better encompassing foot exercises and orthotic therapy on the non-amputated side during the prosthetic rehabilitation period.

## Introduction

Diabetic neuropathy is one of the three major complications of diabetes mellitus. As diabetes progresses, abnormalities in the motor, sensory, and autonomic nervous systems can cause foot deformity, loss of protective sensation, difficulty sweating, and dry skin. The combination of these factors quickly leads to the formation of calluses on the feet and soles, and repeated load-bearing causes bleeding in the deeper layers of the calluses. If a blood flow disturbance, such as peripheral artery disease, is present, the wound does not heal well, and ulceration occurs [1]. Approximately 15-25% of patients with diabetes develop foot ulcers during their lifetime [2]. Therefore, it is essential to detect diabetic neuropathy causing diabetic foot ulcers early and take measures to prevent diabetic lower limb amputations.

The Ipswich Touch Test (IpTT) is a simple and novel method for diabetic peripheral neuropathy. It involves lightly touching or resting the tip of the index finger for one to two seconds on the tips of the first, third, and fifth toes. Neuropathy is present if two or more sites are insensate [3-5]. The IpTT was found to have similar sensitivity, specificity, and operating characteristics as the 10-g monofilament, which is considered the gold standard, when assessed against a vibration perception threshold (VPT) ≥25 V. Sensitivity and specificity for the IpTT against a VPT ≥25 V were 77% and 90%, respectively [4].

The IpTT was originally a screening test for neuropathy performed on both toes in patients with diabetes before amputation and was not intended for lower limb amputation. Therefore, given that the amputated toe does not remain after major amputation, we only performed this test on the non-amputated toes. We call this variation the modified IpTT (mIpTT). We diagnosed neuropathy if a patient had one or more sensory deficits on the non-amputated side.

The prevalence of diabetic neuropathy is estimated to be 26.71%, or between 6% and 51%, among adults with diabetes, depending on the age, duration of diabetes, country, and other factors [6,7]. However, a few reports on the prevalence of neuropathy in patients with diabetes whose one side has undergone lower limb amputation exist. In addition, there are also few reports of prosthetic rehabilitation for diabetic lower limb amputees with neuropathy and orthotic therapy for non-amputated foot.

We accept many patients who have undergone lower limb amputation due to diabetes mellitus in our convalescent rehabilitation ward. Therefore, we are investigating the mIpTT positivity rate in amputees with diabetes and using the results for rehabilitation. Here, we report the mIpTT findings in diabetic lower limb amputees and describe the rehabilitation treatment of individuals with positive results.

## Materials and methods

This retrospective study included lower limb amputees admitted to our convalescent rehabilitation ward for prosthetic rehabilitation between April 2019 and December 2022. We referred to medical records to identify all lower limb amputees during this period and categorized these amputees by the level of amputation. Bilateral lower limb amputees, for whom we could not perform mIpTT, were excluded from this study. We then selected lower limb amputees with diabetes from among these patients. Finally, we divided these lower limb amputees with diabetes by the level of amputation and counted the mIpTT results by gender and age.

We performed the mIpTT for the lower limb amputees with diabetes immediately after hospital admission. The mIpTT involves lightly touching or resting the index finger for one to two seconds on the tips of the first, third, and fifth toes on the non-amputated side (Video 1). We diagnosed neuropathy as present if one or more sites are insensate.

**Video 1:** modified Ipswich touch test The examiner lightly touches the patient's tips of the first, third, and fifth toes with the index finger for one to two seconds.

We examined the medical records to gather information on physical therapy, orthotic therapy, and prosthetic rehabilitation. Specifically, we reviewed the details of the physical therapy performed on the non-amputated foot. In addition, we investigated the type of orthotic structure that the prosthetists and orthotists created during orthotic therapy. In Japan, a prosthetist and orthotist can fabricate both prostheses and orthotics.

This retrospective study was approved by the Ethics Committee of JR Tokyo General Hospital (R04-20). Informed consent was obtained from all the patients using the opt-out method.

## Results

A total of 94 lower limb amputees were identified. Of the 94 lower limb amputees, 49 were transtibial amputees, 28 were transfemoral amputees, three were hip disarticulation amputees, and 14 were bilateral lower limb amputees. Of these amputees, 27 transtibial amputees and nine transfemoral amputees are lower limb amputees with diabetes (Table 1). There were more male than female amputees with diabetes included in this study. The mIpTT was positive in 22 (81%) transtibial and eight (89%) transfemoral amputees (30 in total; 83%). There were no apparent differences in positive rates based on amputation level, gender, or age. Six negative mIpTT patients were present; four of six patients had arteriosclerosis obliterans (ASO) complications, whereas the remaining two had no history of ASO.

**Table 1:** Results of mIpTT AK, above knee amputee; BK, below knee amputee; mIpTT, modified Ipswich Touch Test ^*^The values are given as the number of patients, with the percentage in parentheses.

	No. of Patients	No. of positive mIpTTs^*^
BK	27	22(81%)
Gender		
Male	21	18(86%)
Female	6	4(67%)
Age		
≧60	16	13(81%)
<60	11	9(82%)
AK	9	8(89%)
Gender		
Male	7	6(86%)
Female	2	2(100%)
Age		
≧60	3	3(100%)
<60	6	5(83%)
Total	36	30(83%)
Gender		
Male	28	24(86%)
Female	8	6(75%)
Age		
≧60	19	16(84%)
<60	17	14(82%)

Rehabilitation

**Figure 1:**
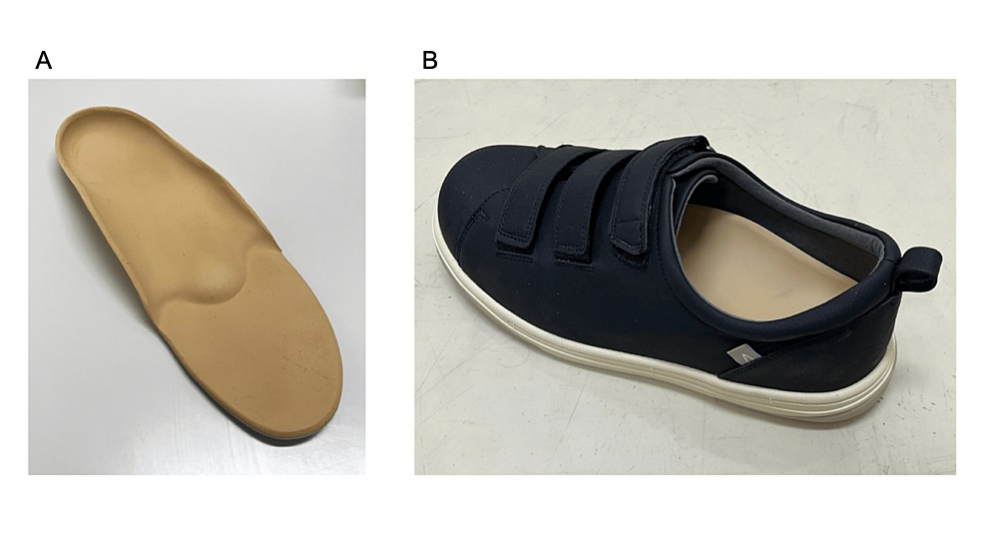
Insole and Specialized shoe (A) Prosthetist and Orthotist made an insole to form transverse and longitudinal arches. (B) This shoe is a size 7E, has a flat heel, and is easy to wear with a prosthetic foot.

**Figure 2:**
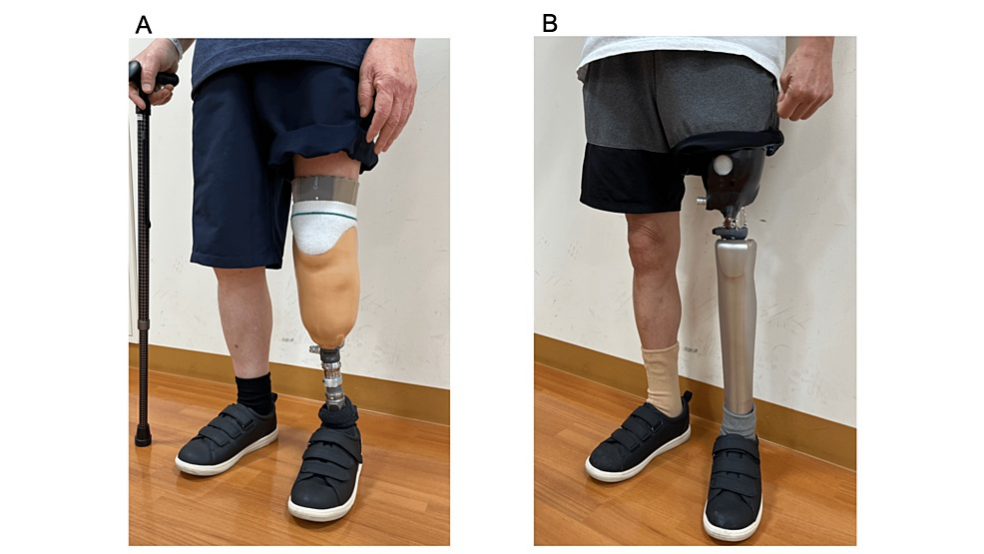
Lower limb prostheses (A) Transtibial prosthesis. (B) Transfemoral prosthesis.

We performed range of motion exercises of the toes and ankle joints and towel-gathering (Video 2) and marble-grasping (Video 3) exercises with the toes in patients diagnosed with diabetic neuropathy based on the mIpTT results. In addition, we prescribed insoles (Figure 1A) as orthotic therapy. The prosthetists and orthotists fabricated insoles with longitudinal and transverse arch structures to help improve the shape of the diabetic foot. They also fabricated partially soft insoles to relieve pressure on any ulcers on the patient's feet, depending on the patient. Furthermore, we had the patients purchase shoes suitable for diabetic feet (Figure 1B). This shoe has the following features: a large boll girth (7E), the ability to insert an insole, a low heel pitch (zero), and a large forefoot opening that makes it easy for the prosthetic foot to wear. Finally, we prescribed all patients lower limb prostheses (Figure 2).

**Video 2:** Patient performing towel-gathering exercise The patient is trying to grasp a towel on the floor with his toes but cannot do so firmly.

**Video 3:** Patient performing marble grasping exercise Diabetic neuropathy often prevents patients from grasping marbles with their toes.

## Discussion

Individuals with diabetes are at risk of lower limb amputation. Diabetes is the most common cause of non-traumatic amputation, with approximately 1% of cases resulting in lower limb amputation [8]. Diabetic lower limb amputations account for 68.6% of all lower limb amputations [9]. The incidence of major lower limb amputations (e.g., amputations proximal to the ankle joint) in Japan was also 10 times higher in people with diabetes than those without [10]. Approximately two-thirds of nontraumatic amputations performed in the USA are due to diabetic foot ulcers [11]. For individuals with active diabetic ulcers, the healing rate is between 65% and 75% for those attending primary care centers. However, approximately 15-20% of individuals with ulcers undergo amputation, depending on the duration of follow-up [12].

The incidence of contralateral amputation among amputees with diabetes was 20.5% over five years [13]. Lower limb amputees with diabetes are at a high risk of subsequent amputation on the non-amputated side. These findings are also supported by the high prevalence of diabetic neuropathy on the non-amputee side (83%) in this study.

Preventing diabetic foot ulcers progressing from diabetic neuropathy is essential to prevent diabetic lower limb amputation. This task is more critical for patients with a lower limb amputation on one side. Therefore, when lower limb amputees with diabetes are admitted to a convalescent rehabilitation ward for prosthetic rehabilitation, we must diagnose an existing neuropathic complication and initiate an immediate therapeutic intervention for the foot on the non-amputated side. These interventions include exercise therapy for the toe and ankle joint and foot orthotics. In addition, prosthetic rehabilitation is crucial for teaching patients to use the lower limb prosthesis correctly and avoid overburdening the non-amputated side.

Evidence has demonstrated that foot exercises can address the risk factors for diabetic foot ulcers, such as low foot-ankle joint mobility and strength [14-17]. In addition, foot-ankle flexibility and resistance exercises can reduce the recurrence of plantar foot diabetic ulcers and improve diabetic neuropathy [18]. Physiotherapy must also be provided to patients because of their impaired balance ability due to diabetic neuropathy. Poor balance places excessive stress on the non-amputated foot, increasing the risk of diabetic foot ulcers. Exercise interventions, such as strength training, balance training, gait training, Tai Chi, and combined exercise regimens, can improve balance capacity in patients with type 2 diabetes mellitus [19].

Prescriptive shoes with orthotic inserts are often prescribed to prevent recurrent ulceration [20]. Furthermore, prescriptive shoes with customized insoles based on pressure analysis showed a significant reduction in the risk of reulceration [21,22]. This finding demonstrates the benefits of insoles and customized shoes for foot ulcer prevention in lower limb amputees with diabetes. Therefore, we prescribed insoles to this population and had them purchase shoes more suitable for their condition. We ordered insoles with designs that supported the transverse and longitudinal arches and softened the areas of the sole that encountered the calluses from the prosthetists and orthotists. We recommend that patients choose shoes appropriate for their foot length and circumference. Figure 1 shows a shoe that patients in our hospital often purchase. This shoe has the following features: a large boll girth (3E or 7E), the ability to insert an insole, a small heel pitch, and a large forefoot opening, making it easy for the prosthetic foot to wear. We recommend that prosthetic users choose shoes with a low heel pitch; therefore, no difference in walking comfort is observed between indoor and outdoor use, that is, with and without shoes.

Four of the six mIpTT-negative patients had severe ASO and were treated with endovascular therapy of the lower extremity arteries. We considered these cases more affected by insufficient blood flow due to ASO than diabetes. Two mIpTT-negative patients had diabetes without ASO, with the reasons for their amputation being necrotizing fasciitis in one and difficulty in controlling infection following foot ulceration and toe osteomyelitis in the other. These results indicate that even without neuropathy, diabetes mellitus can lead to amputation if complicated by a rapidly progressing infection due to poor glycemic control.

Limitations

Although a longer duration of diabetes and an extended period of inadequate treatment can predict the high incidence of neuropathy, determining the exact history of diabetes in this study was difficult because many lower limb amputees with diabetes fail to continue diabetes treatment. The time of disease onset and duration of treatment are often unknown. In addition, while the original IpTT was performed on both toes of the patient, we performed this test on only one foot because the patients were amputees; however, this test was not validated when performed on only non-amputated toes. We must verify whether the sensitivity and specificity are reasonable in mIpTT compared to 10g-monofilament or IpTT. Although all subjects in this study were diabetic lower limb amputees, these patients were eligible for lower limb prostheses, and those not eligible for the prostheses were exclusive. However, even if these patients who are not eligible for lower limb prostheses are included, they are likely to have more severe diabetes. Therefore, mIpTT positive rate would not be lower than our study. The rehabilitation described in this study is what we are currently working on, and it is not clear at this time if this approach can prevent contralateral amputation in patients. Therefore, continued follow-up is needed.

## Conclusions

Lower limb amputees with diabetes may have a high rate of neuropathy in non-amputated lower limbs. Therefore, we believe that physicians and physical therapists in charge of the rehabilitation treatment of diabetic leg amputees should utilize the mIpTT, a simple screening test for diabetic neuropathy, and actively implement foot and ankle exercises and prescribe orthotics, such as insoles and shoes to protect the non-amputated lower limbs, along with prosthetic rehabilitation.
